# Effect of Aplidin in acute lymphoblastic leukaemia cells

**DOI:** 10.1038/sj.bjc.6601130

**Published:** 2003-08-12

**Authors:** E Erba, M Serafini, G Gaipa, G Tognon, S Marchini, N Celli, D Rotilio, M Broggini, J Jimeno, G T Faircloth, A Biondi, M D'Incalci

**Affiliations:** 1 Flow Cytometry Unit, Department of Oncology, Istituto di Ricerche Farmacologiche ‘Mario Negri’, Via Eritrea, 62-20157 Milan, Italy; 2 M Tettamanti Research Center, Pediatric Clinic, University of Milan Bicocca, Via Donizetti 106, 20052 Monza, MI, Italy; 3 Consorzio Mario Negri Sud, Via Nazionale, 66030 Santa Maria Imbaro, CH, Italy; 4 Pharma Mar, S.A., Poligono Industrial La Mina, Avda. de Los Reyes 1, 28770 Colmenar Viejo, Madrid, Spain; 5 Pharma Mar USA, Inc., 320 Putnam Avenue, Cambridge 02139-4616, MA, USA

**Keywords:** marine natural compounds, apoptosis, cell cycle, stroma-supported immunocytometric assay

## Abstract

The cytotoxic effect of Aplidin was investigated on fresh leukaemia cells derived from children with B-cell-precursor (BCP) acute lymphoblastic leukaemia (ALL) by using stromal-layer culture system and on four cell lines, ALL-PO, Reh, ALL/MIK and TOM-1, derived from patients with ALL with different molecular genetic abnormalities. In ALL cell lines Aplidin was cytotoxic at nanomolar concentrations. In the ALL cell lines the drug-induced cell death was clearly related to the induction of apoptosis and appeared to be p53-independent. Only in ALL-PO 20 nM Aplidin treatment caused a block of vascular endothelial growth factor (VEGF) secretion and downregulation of VEGF-mRNA, but Aplidin cytotoxicity does not seem to be related to VEGF inhibition since the sensitivity of ALL-PO cells to Aplidin is comparable to that observed for the other cells used. Aplidin induced a G_1_ and a G_2_ M block in ALL cell lines. In patient-derived leukaemia cells, Aplidin induced a strong cytotoxicity evidenced in a stroma-supported immunocytometric assay. Cells from children with genetic abnormalities such as t(9;22) and t(4;11) translocations, associated with an inferior treatment outcome, were sensitive to Aplidin to the same extent as that observed in other BCP-ALL cases. Aplidin exerted a strong cell killing effect (>88%) against primary culture cells from five relapsed ALL cases, at concentrations much lower than those reported to be achieved in plasma of patients receiving Aplidin at recommended doses. Taken together these data suggest that Aplidin could be a new anticancer drug to be investigated in ALL patients resistant to available therapy.

Acute lymphoblastic leukaemia (ALL) is the most common form of cancer in children. It arises in bone marrow from malignant B-lymphoid progenitors. Among the distinguishing cellular features of ALL, clonal abnormalities can be identified in approximately 65–70% of cases. Acute lymphoblastic leukaemia cells are sensitive to several currently used drug treatments, but in approximately one-third of the children, the disease recurs during, or following, therapy (Pui, 2000; Pui *et al*, 2001). Therefore, the identification of new antileukaemia drugs that are effective against ALL, particularly following a relapse, may help further improvement of treatment. Acute lymphoblastic leukaemia-derived primary cells are being used to improve the sensitivity of *in vitro* methods to measure new drug effectiveness–the goal of the work is presented here.

Aplidin (dehydro-didemnin B) is a marine depsipeptide isolated from the Mediterranean tunicate *Aplidium albicans*. It has shown activity against both human haematological and solid tumour cell lines growing *in vitro* (Urdiales *et al*, 1996; Lobo *et al*, 1997; Depenbrock *et al*, 1998; Geldof *et al*, 1999), *in vivo* murine B16 melanoma and in several human tumours xenografts (Faircloth *et al*, 1995, 1996).

Aplidin inhibits the progression of cells from G_1_ to the S phase of the cell cycle (Crampton *et al*, 1984). Recently, a G_2_ block was described for Aplidin in human leukaemia Molt-4 cell line (Erba *et al*, 2002). Inhibition of protein synthesis via GTP-dependent elongation factor 1-alpha *in vitro* (Crews *et al*, 1994), DNA and RNA syntheses in different cell lines (Crampton *et al*, 1984; Sakai *et al*, 1996) and inhibition of ornithine decarboxylase have been reported for Didemnins as well as for Aplidin (Urdiales *et al*, 1996; Erba *et al*, 2002).

In phase I and phase II clinical studies that are in progress, no neutropenia but a moderate lymphopenia has been observed (Raymond *et al*, 2000; Armand *et al*, 2001; Ciruelos Gil *et al*, 2002).

In this study, we assess the cytotoxic effect of Aplidin on fresh leukaemia cells derived from children with B-cell-precursor (BCP) ALL by using stromal-layer culture system, established by Campana and co-workers (Manabe *et al*, 1992; Campana *et al*, 1993, 1999; Kumagai *et al*, 1996). Aplidin effectiveness was also evaluated by assessing the cytotoxicity and the induction of apoptosis in four human leukaemic cell lines, ALL-PO, Reh, ALL/MIK and TOM-1, derived from patients with ALL with different molecular genetic abnormalities.

## MATERIALS AND METHODS

### Cell lines

The leukaemic cell lines, Reh, ALL/MIK, TOM-1 (Rosenfeld *et al*, 1977; Okabe *et al*, 1987; Higa *et al*, 1994) and ALL-PO (Gobbi *et al*, 1997), were derived from BCP-ALL patients and are all characterised by particular chromosomal translocations that are representative of the molecular genetic abnormalities most frequently found in childhood: two Philadelphia-positive cell lines TOM-1 and ALL/MIK cells with a t(9;22), Reh cells with a t(12;21) and ALL-PO cells with a t(4;11).

Cells were grown in an RPMI 1640 culture medium (Sigma-Aldrich, St Louis, MI, USA) with 10% Hyclone FBS (Hyclone Laboratories, Inc., Logan, UT, USA) and 4 mM glutamine at 37°C in a humidified atmosphere containing 5% CO_2_ in T25 cm^2^ tissue culture flasks (IWAKY, Bibby Sterilin, Staffordshire, UK).

### Stroma-supported cultures of ALL cells

Leukaemic bone-marrow (BM) samples were collected from 14 patients with BCP-ALL aged less than 1–14 years (median, 6 years) either at the time of diagnosis (nine patients) or at relapse (five patients). The institutional review board approved this study and informed consent was obtained from patients and their guardians.

In all cases, more than 80% of the blasts were positive for CD19, class II antigens, terminal deoxynucleotidyl transferase (TdT), and lacked surface immunoglobulins (sIg). Karyotype features were determined by conventional banding techniques. Other clinical features of the patients are listed in Table 1

**Table 1:** Clinical features of the patients

**Case (no)**	**Age (years)**	**Sex**	**WBC (l^−1^)**	**Phenotype**	**DNA index**	**Karyotype**	**Follow-up**
1	11	F	21 × 10^9^	cALL	1,00	46,XX	+8 CR
2	2	M	11 × 10^6^	cALL	1,20	53, XY,+4,+6,+8,+12,+20+21+21+21	+8 CR
3	13	F	14 × 10^6^	Pre-Pre-B-ALL	1,00	46,XX	+5 CR
4	4	F	11 × 10^6^	cALL	1,06	50,XX,+21[c]	+5 CR
5	<1	F	13 × 10^6^	Pre-B-ALL	1,00	46,XX,+8,+19,+22	+4 CR
6	3	M	7,9 × 10^6^	cALL	1,00	47,XY,del(14q21),+marker	+5 CR
7	4	M	350 × 10^6^	cALL	1,00	46,XY t (9;22)	Resistant
8	10	F	27 × 10^6^	cALL	1,21	58,XX,+ × ,+1,+3,+8,+14,+16,+18,21,+19,+19,+21,+21,+21/46,XX	+30 CR
9	9	M	140 × 10^6^	Pre-pre-B ALL	1,00	46,XY t (4;11)/47,XY	+38 CR
10^a^		F	11 × 10^6^	cALL	ND	ND	Exitus
11^a^	7	M	13 × 10^6^	Pre-B ALL	1,00	46, XY	Exitus
12^a^	5	F	132 × 10^6^	LLA B mature	1,00	No mitosis	+2 TMO
13^a^	3	M	58 × 10^9^	cALL	1,00	ND	Exitus
14^a^	9	M	35 × 10^9^	Null ALL	ND	46,XY	Exitus

a =relapse. ND=not determined.

.

Mononuclear cells (MNC) were obtained by centrifugation on a density gradient using Ficoll-Paque (Pharmacia LKB, Uppsala, Sweden). After washing with phosphate-buffered saline (PBS) ALL samples were cryopreserved in RPMI-1640 medium, with 50% heat-inactivated FBS Hyclone and 10% dimethyl sulphoxide (DMSO) and were stored in liquid nitrogen before use in the *in vitro* studies.

Previously frozen leukaemic cells were cultured and only cultures that had greater than 90% cell viability by trypan blue dye exclusion were used.

Bone marrow stromal layers were prepared as previously described by Campana and co-workers (Manabe *et al*, 1992; Campana *et al*, 1993, 1999). Briefly, normal BM MNC were resuspended in RPMI-1640 that contained 10% FCS, 1 *μ*M hydrocortisone, 2 mM L-glutamine, and 1% penicillin–streptomycin. Cells were incubated at 37°C in 5% CO_2_ and 90% humidity in T 25 cm^2^ tissue culture flasks (IWAKY) and were fed every 7 days by replacing 50% of the supernatant with identical fresh medium.

After formation of confluent layers, cells were detached by trypsin, washed once with RPMI-1640 with 10% FBS and resuspended in fresh stroma culture medium. Cells were seeded in 96-well flat-bottom plates at 3 × 10^4^ cells well^−1^.

### Drug treatment on cell lines

Aplidin was kindly supplied by PharmaMar SA (Madrid). The effect of drug treatment on cell lines was evaluated by a standard growth inhibition assay.

Leukaemia cells in the exponentially growing phase were treated for 1 h with different concentrations of Aplidin. After treatment, cells were washed with PBS and incubated with fresh drug-free medium; viable cells number was estimated by means of a Coulter Counter (Beckman Coulter Corp., Hialeath, FL, USA) at different time intervals after drug-washout.

### Drug treatment on leukaemic BM cells

To determine the cytotoxicity of Aplidin in patient samples, we first established the cells in culture. The media from the BM stroma was removed and the adherent cells were washed seven times with AIM-V serum-free tissue culture medium (Gibco BRL). Resuspended leukaemia cells in AIM-V medium were aliquoted as 3 × 10^5^ cells on the stromal layers. Blast cells from individual patients were exposed for 7 days at 37°C in 5% CO_2_ and 90% humidity to different Aplidin concentrations in log increments that ranged from 0.005 to 5 nM.

### Cell cycle

#### Monoparametric DNA analysis

Exponentially growing leukaemic cells were treated for 1 h with 0, 5, 10, and 20 nM Aplidin. After treatment, the drug-containing medium was removed, the cells were washed with PBS and were placed in fresh medium. At different time intervals after drug-washout, the number of cells was evaluated by a Coulter Counter and the cells were fixed in 70% ethanol and kept at 4°C before DNA staining (Erba *et al*, 2002).

#### Biparametric BrdUrd/DNA analysis

During the last 15 min of drug treatment, 20 *μ*M bromodeoxyuridine (BrdUrd) was added to the cells. After treatment the drug-containing medium was removed, the cells were washed twice with PBS and fresh medium was provided. After 1 h treatment and at different time intervals after drug-washout, control and treated cells were fixed in 70% ethanol and stored at 4°C before staining. With this protocol it was also possible to obtain a distinct evaluation of cell cycle perturbations in cells which were in S phase (BrdUrd-positive cells), G_1_ phase or G_2_ M phase (BrdUrd-negative cells) during 1 h 10 nM Aplidin exposure (Erba *et al*, 2002).

### Detection of apoptosis and caspase-3 activation in ALL cell lines

At different time intervals after drug-washout, the cells were fixed in 70% ethanol for terminal-dUTP-transferase (TdT) or in 1% paraformaldehyde for caspase analysis and stored at 4°C. The fixed cells were washed in cold PBS and incubated in 50 *μ*l of TdT and FITC-conjugated dUTP deoxynucleotides solution (Roche Diagnostic SpA, Milan, Italy) or PE-conjugated rabbit anti-active caspase-3 (Becton Dickinson, San José, CA, USA) and analysed by flow cytometry (Erba *et al*, 2002).

### Assessment of cytotoxicity and apoptosis on leukaemic BM cells

Before and after culturing on stroma, the ALL cell recovery and phenotype were determined as previously described (Manabe *et al*, 1992; Campana *et al*, 1993). Briefly, at termination of the cultures, cells were passed through a 19-gauge needle to disrupt clumps formed by stromal cells, washed with PBS that contained 0.2% bovine serum albumin and 0.2% sodium azide (PBSA) and incubated with Leu-12-PE (anti-CD19, Becton Dickinson, San Jose, CA, USA). After two washes in PBSA, the cells were resuspended in 0.5% paraformaldehyde and counted using a FACScan instrument and Cell Quest software (Becton Dickinson). Using the light-scatter dot plot, which depicts cell size and granularity, we identified the area where the vast majority of viable leukaemia cells were located at the beginning of cultures. This area was delimited with an electronic gate, which subsequently was used to enumerate leukaemia cells at the end of culture. The number of nonapoptotic lymphoid cells detected within a 30-s interval was corrected for the percentage of CD19+ cells present. The cell counts of drug-treated and control samples were compared to calculate the percentage of viable cells that remained after drug treatment. The following formula was used to calculate relative cell recovery after drug treatment: (no. cells recovered with drug)/(no. of cells recovered without drug) × 100.

### VEGF RNAse protection

Exponentially growing cells were treated for 1 h with Aplidin (20 nM) and total RNA purified with the TRIZOL reagent (Gibco BRL) at 1, 6 and 24 h after drug-washout. VEGF and VEGFR-1 mRNA were measured by RNase protection assay using a commercially available kit (Becton Dickinson).

### ELISA assay

The assay, aimed at evaluating the concentration of VEGF-A in the medium of cells, was performed on 96-well plates coated with an anti-VEGF antibody (Quantikine kit, R&D Systems Europe, Oxon, UK). Standards of VEGF protein ranging from 1000 to 31.2 pg ml^−1^ were prepared after reconstituting VEGF standard with 1 ml of calibrator diluent. To each well, 50 *μ*l of Assay Diluent and 200 *μ*l of medium (or standard) were added; after 2 h of incubation, wells were washed three times with wash buffer and 200 *μ*l of VEGF-conjugated were added. After 2 h, wells were washed three times and 200 *μ*l of Substrate Solution was added. After 20 min, 50 *μ*l of stop solution was added to each well and optical density was evaluated by means of a plate-reader spectrophotometer (Labsystem Multiskan, Dasit, Italy) at 540 nm.

### Liquid chromatography–tandem mass spectrometry analysis

Liquid chromatography–tandem mass spectrometry (LC–MS/MS) analyses of Aplidin in medium were performed using a method similar to that described for rat plasma (Celli *et al*, 1999).

## RESULTS

### Cytotoxicity, apoptosis and cell cycle perturbations induced by Aplidin on ALL cell lines

Figure 1A

**Figure 1:**
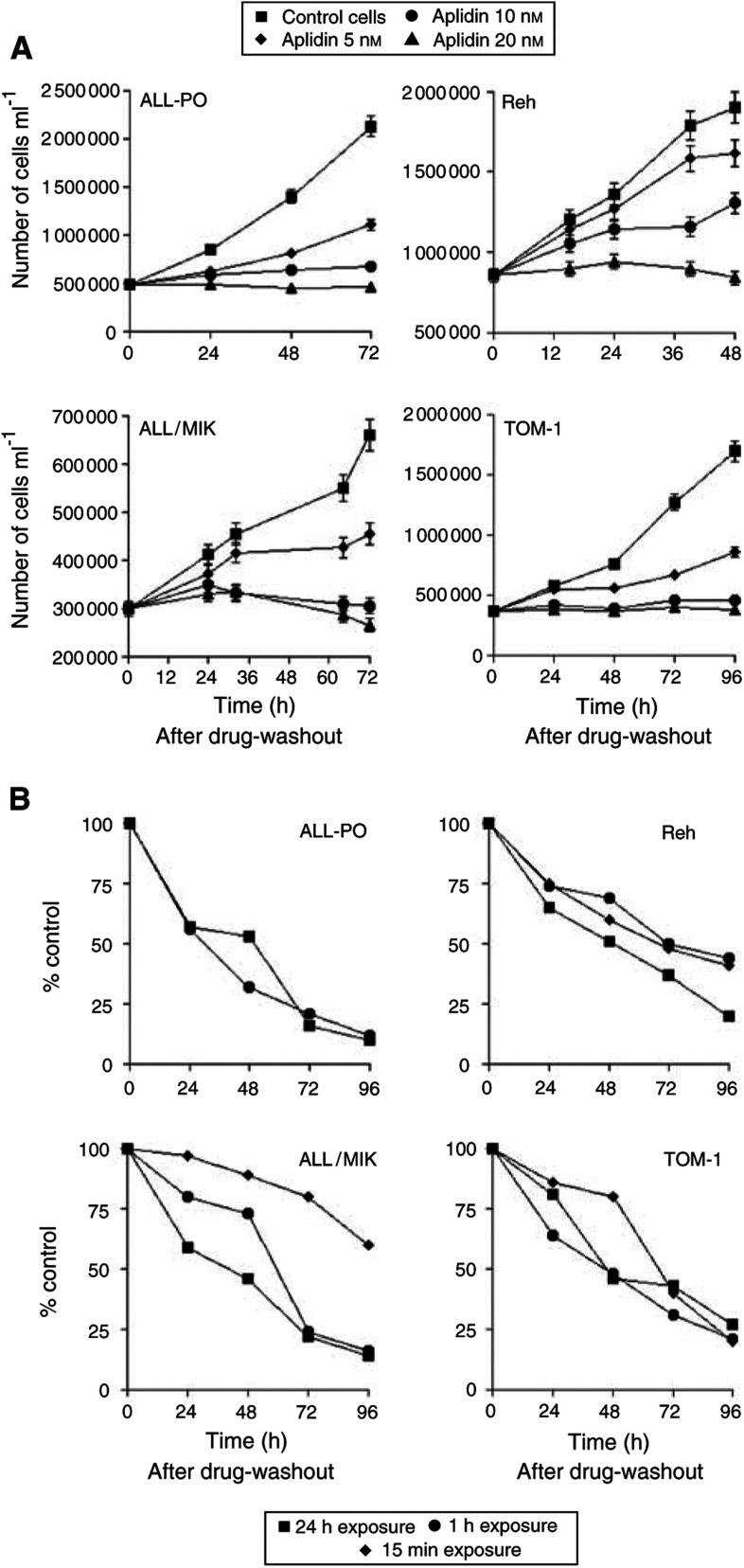
Effect of 1 h (**A**) or 15, 60 min 24 h (**B**) Aplidin exposure on cell growth evaluated at different time intervals after treatment and drug-washout on ALL-PO, Reh, ALL/MIK and TOM-1. Each point is the mean of three replicates; bars respresent the standard deviation.

shows the growth inhibitory effect of 1 h treatment with different concentrations of Aplidin on different cell lines. Aplidin was active at nanomolar concentrations in all the cell lines and the cytotoxic effect was dose dependent. An irreversible growth inhibitory effect was observed after 1 h using 10 nM Aplidin treatment in All-PO, ALL/MIK and TOM-1 cells while in Reh cells Aplidin induced an irreversible growth inhibitory effect, but only at 20 nM. To assess the sensitivity of the leukaemic cell lines to Aplidin at different exposure times, cells were incubated with 20 nM Aplidin for 15 min, 1 or 24 h. As shown in Figure 1B the cytotoxicity against ALL-PO, Reh and TOM-1 cells was similar regardless of the incubation times. In contrast, ALL/MIK cells with 15 min of exposure to Aplidin survived longer than ALL/MIK cells after more prolonged treatments. Yet, an optimal effect was achieved by 1 h since similar effects were also seen at 24 h (Figure 1B).

The lack of increased cytotoxicity of Aplidin beyond 1 h compared to a short exposure time might have been due to a rapid decomposition of the peptide under cell culture conditions. However, this was not found to be the case. After 24 h incubation of Aplidin in medium at 37°C, 80% of the drug was detected as unchanged by HPLC–MS (data not shown).

### Cell cycle perturbations induced on ALL cell lines

Figure 2

**Figure 2:**
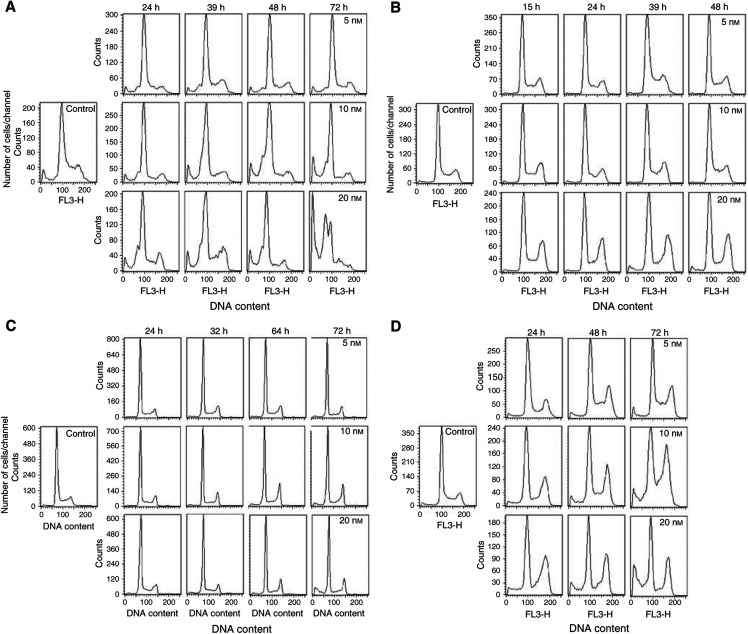
Cell cycle phase perturbations induced on (**A**) ALL-PO, (**B**) Reh, (**C**) ALL/MIK and (**D**) TOM-1 cells treated for 1 h with 0, 5, 10 or 20 nM Aplidin. Monoparametric DNA flow cytometric analysis were performed at different time intervals after drug-washout.

shows the effects on the cell cycle phase distribution caused by 1 h exposure with different concentrations of Aplidin in the different leukaemic cell lines evaluated at different time intervals after drug-washout. We found that in all the ALL cell lines Aplidin caused a block of the cells in the G_1_ phase of the cell cycle. In Reh, ALL/MIK and TOM-1 cells Aplidin induced also a G_2_ block.

**Figure 3:**
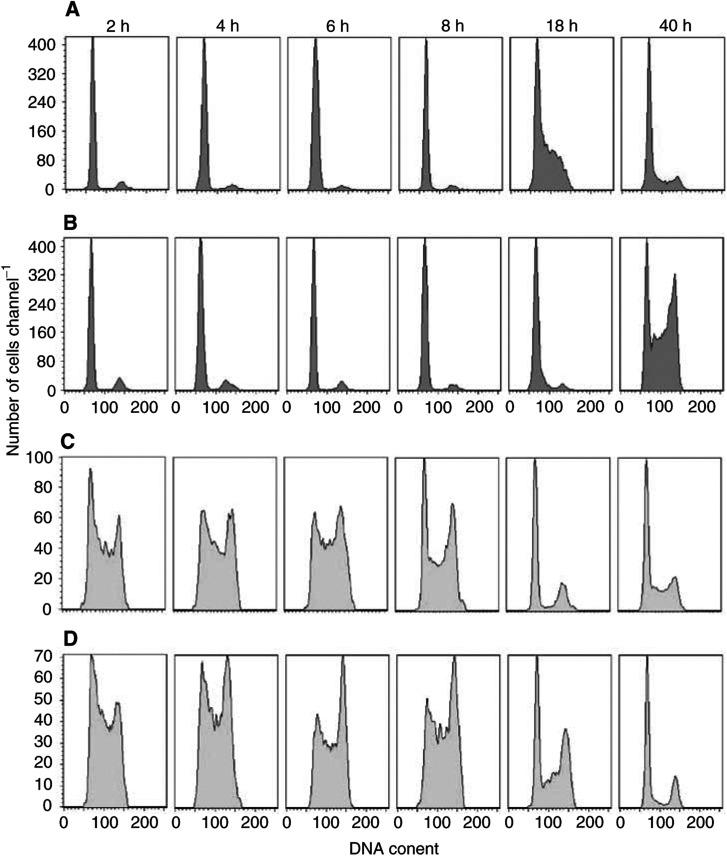
Biparametric BrdU/DNA analysis performed in Reh cells treated for 1 h with 10 nM Aplidin. During the last 15 min of drug treatment 20 *μ*M BrdU was added to the cells, then the cells were washed with PBS and drug-free medium was provided. The flow cytometric analysis were performed at different time intervals after drug-washout. (**A**) DNA histograms of BrdU-negative control cells; (**B**) DNA histograms of BrdU-negative Aplidin-treated cells; (**C**) DNA histograms of BrdU-positive control cells; (**D**) DNA histograms of BrdU-positive Aplidin-treated cells.

To better characterise the cell cycle phase perturbation induced by Aplidin the biparametric BrdU/DNA flow cytometric analysis were performed at different time intervals after drug-washout. Figure 3

reports, as an example, the DNA histograms of BrdU-negative and BrdU-positive cells obtained in Reh cells. Aplidin was found to delay those cells that were in the G_1_ phase (BrdU-negative cell population) during drug treatment from entering S phase. At 40 h after drug-washout, the BrdU-negative cells were accumulated in G_1_ and in G_2_. The cells that were in S phase (BrdU-positive cells) during Aplidin treatment progressed throughout this phase of the cell cycle more slowly than control cells. At 18h and UP 40h after; drug-washout, the BrdU-positive cells were accumulated in G_1_ and in G_2_.

### Aplidin-induced apoptosis on ALL cell lines

**Figure 4:**
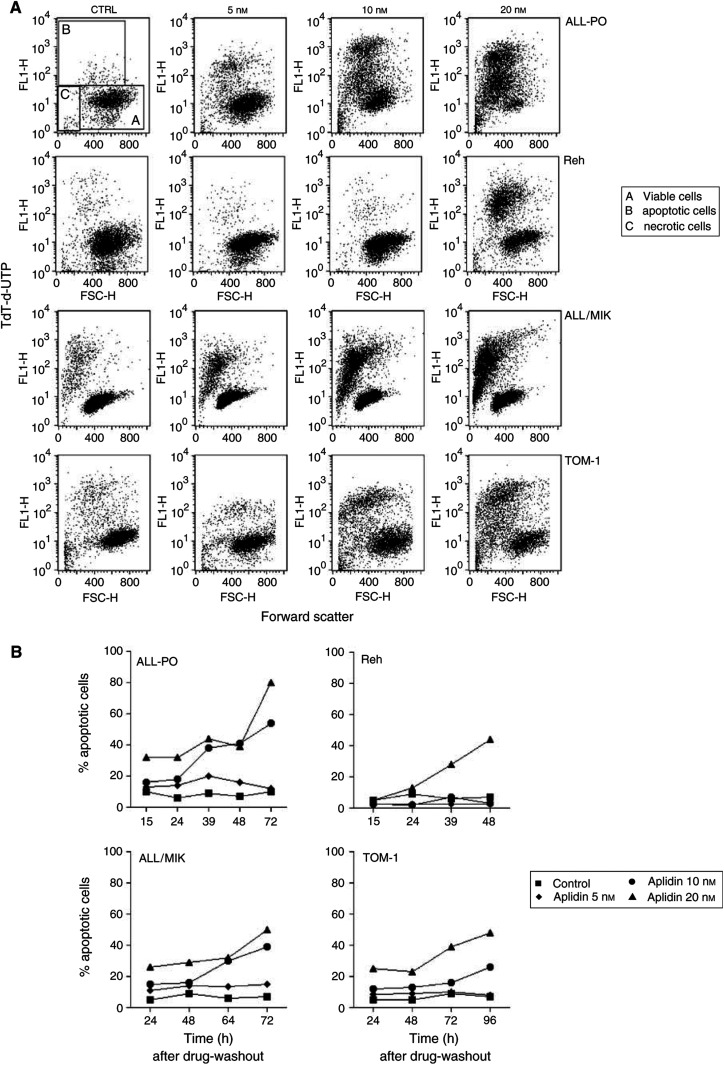
(**A**) Detection of apoptosis in ALL cells by TdT-dUTP flow cytometric analysis. Cells were treated with different concentrations of Aplidin and the biparametric FSC/TdT-dUTP analysis were performed after 72 h after drug-washout. (**B**) Percentage of apoptotic cells evaluated by TdT-dUTP flow cytometric analysis. Cells were treated with different concentrations of Aplidin and biparametric FSC/TdT-dUTP analysis were performed at different time intervals after drug-washout.

**Figure 5:**
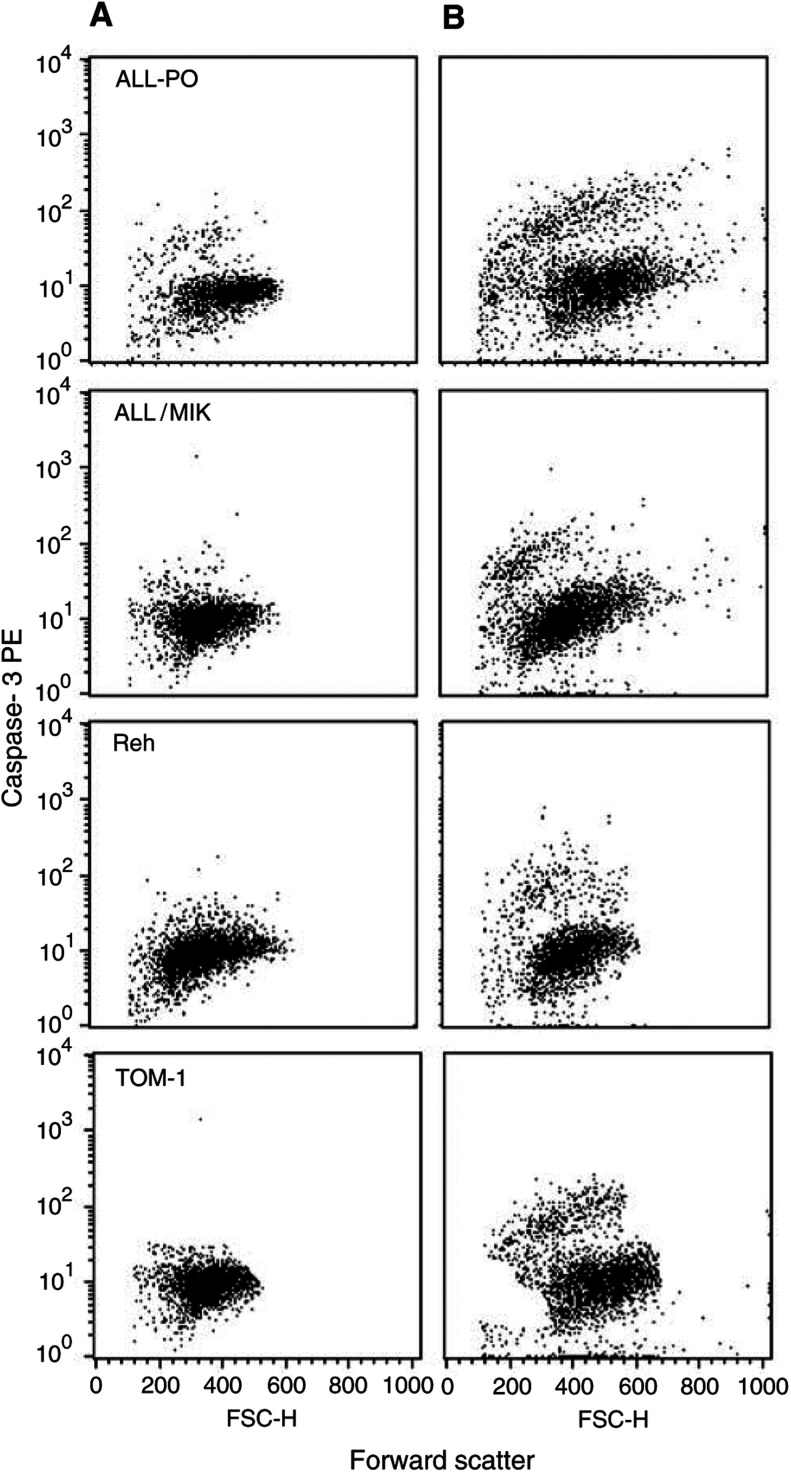
Detection of active caspase-3 in ALL cells by flow cytometric analysis. Cells were treated with different concentrations of Aplidin and the biparametric FSC/caspase-3 analysis was performed at different times after drug-washout. In the figure are reported the analysis performed at 24 h after drug-washout. (**A**) control cells; (**B**) 20 nM Aplidin-treated cells.

It has been reported that Aplidin acts *in vitro* by inducing apoptosis on different cells lines (Grubb *et al*, 1995; Erba *et al*, 2002). Aplidin induced apoptosis in all the cell lines used, as clearly seen by morphological examination by means of sulforhodamine/Dapi staining (data not shown). The level of apoptotic cells was different between the four cell lines as shown in Figure 4A

, which shows an example of the TdT-dUTP flow cytometry analysis, and in Figure 4B, where the percentages of the fraction of apoptotic cells found at different times after drug-washout are reported. In Reh cells, Aplidin induced apoptosis only at the concentration of 20 nM. In ALL-PO, ALL/MIK and TOM-1 the amount of apoptotic cells increased dramatically when the cells were exposed to 10 or 20 nM Aplidin. In All-PO cells at 72 h after drug-washout 80% of the cells treated with 20 nM of Aplidin were apoptotic. As previously reported on other cell type (Garcia-Fernandez *et al*, 2002), Aplidin was found to induce apoptosis in a caspase-3-dependent manner (Figure 5

).

### Modulation of VEGF secretion by Aplidin on ALL cell lines

**Figure 6:**
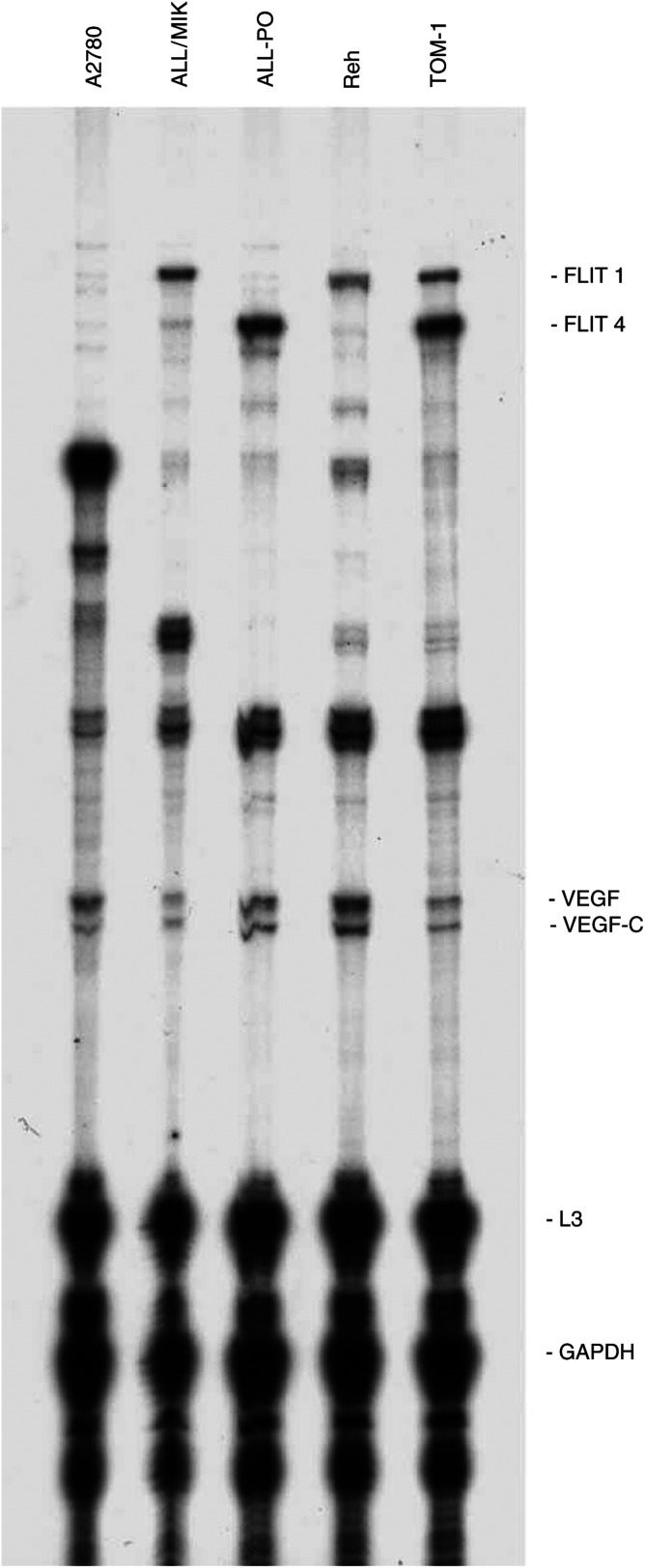
Autoradiography of a typical RNAse protection assay on the four different human leukaemic cell lines. Human ovarian cancer A2780 cells were used as an internal control.

**Figure 7:**
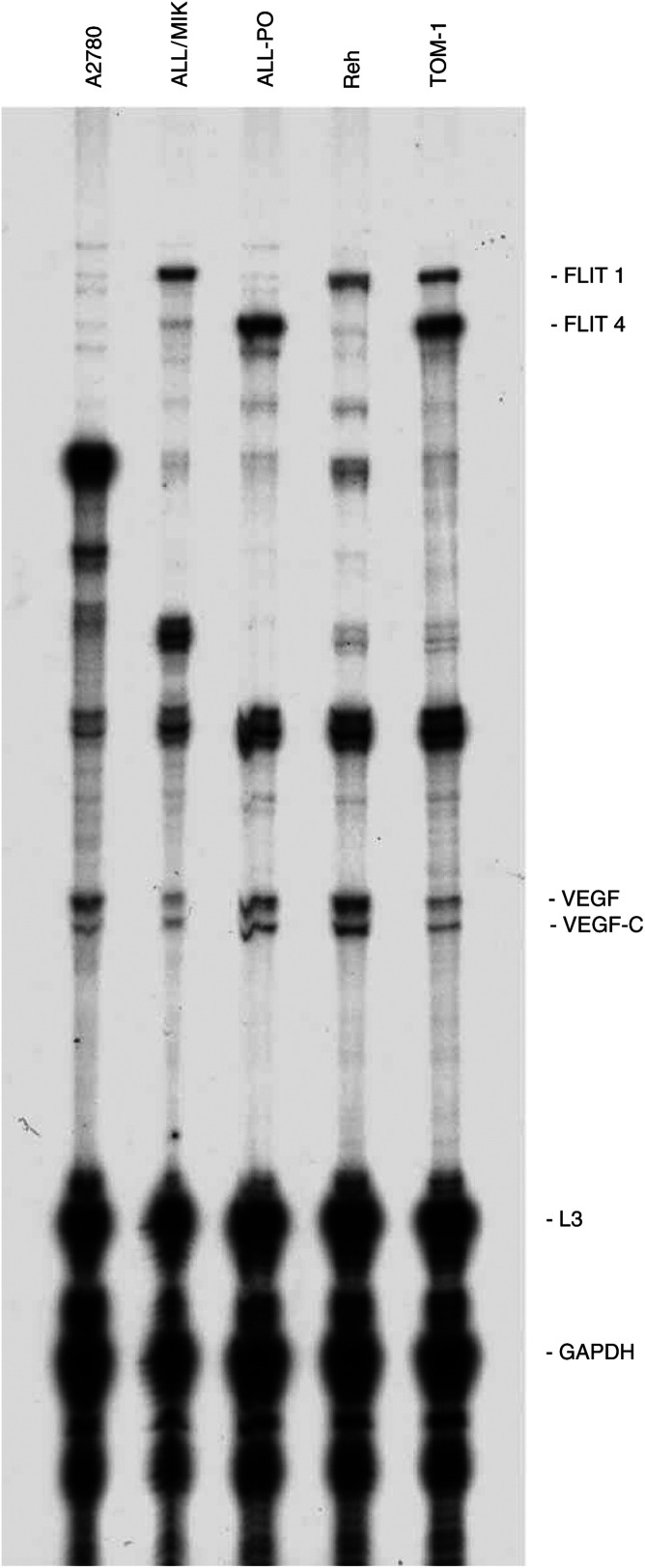
Autoradiography of RNAse protection assay on ALL-PO cells treated with 20 nM Aplidin performed at different time intervals after drug-washout.

**Figure 8:**
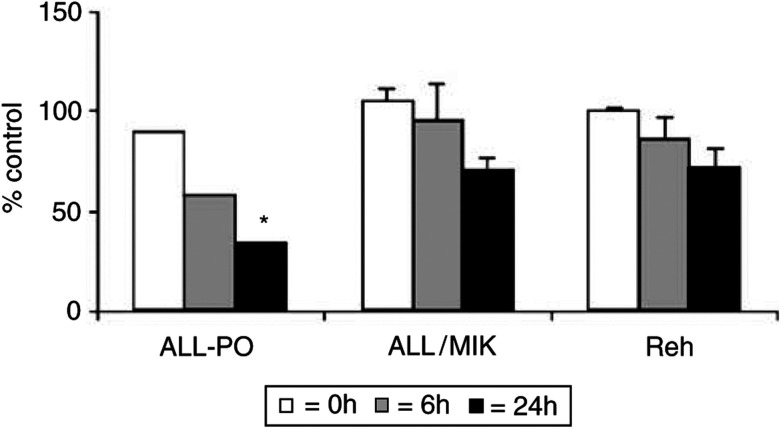
Vascular endothelial growth factor mRNA levels in human leukaemic ALL-PO, ALL-MIK and Reh cell lines treated with 20 nM Aplidin and performed at different time intervals after drug-washout. Data have been obtained by densitometric analysis and expressend as % of control untreated cells. Each column represents the mean of three independent replicates. The bars represent s.d. ^*^=*P*<0.05 (Duncan test).

It has been reported by our group that Aplidin causes a strong block of VEGF secretion in Molt-4 cells with a subsequent downregulation of the transcription of VEGF and of its receptor VEGFR1 (Broggini *et al*, 2003). To test the hypothesis that Aplidin could exert its activity in ALL cell lines by inhibiting the VEGF/VEGFR-1 autocrine loop, we used an RNAse protection assay in each cell line. As shown in Figure 6

, all the cell lines used expressed, at different levels, the VEGF mRNA and its receptors. ALL/MIK and Reh expressed the flt-1 receptor, ALL-PO only the flt-4 receptor while TOM-1 expressed both of them. Data reported in Figure 7

show that after different times from drug-washout, Aplidin downregulated the level expression of VEGF mRNA in ALL-PO cells. When tested in the other cell lines this observation was not further confirmed as shown in Figure 8

**Figure 9:**
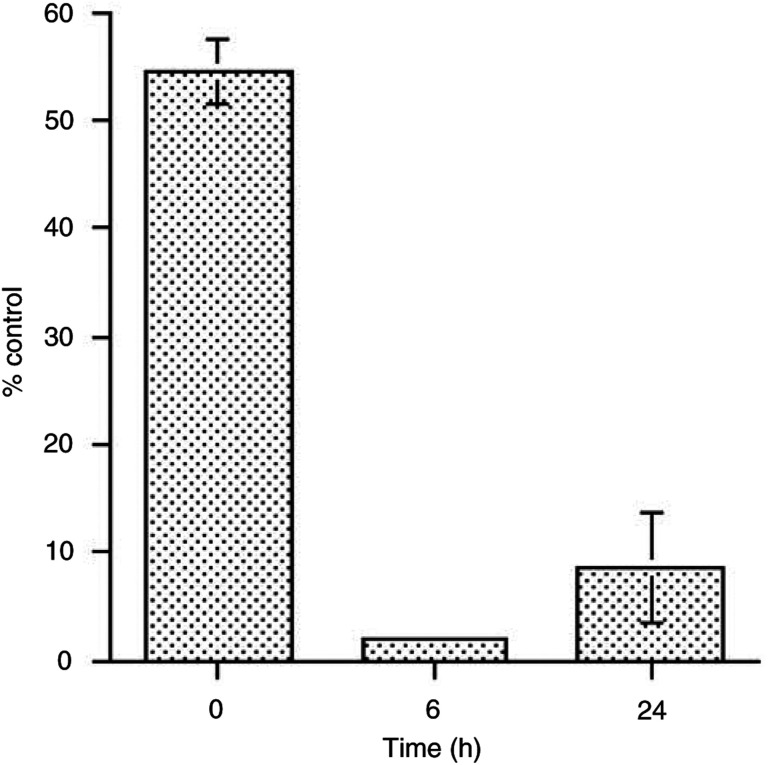
Vascular endothelial growth factor-A concentration in the medium of ALL-PO cells treated for 1 h with 20 nM Aplidin and evaluated at 0, 6 and 24 h after drug-washout. The values express the % of VEGF concentration in the medium of treated cells with regard to control cells.

. By using ELISA assay, we tested the level of VEGF secretion in the four leukaemia cell lines used and found that only ALL-PO secrete detectable amounts of the growth factor. Therefore, we evaluated whether a block in VEGF secretion in ALL-PO cells occurs as previously seen in Molt-4 cells (Broggini *et al*, 2003). We treated ALL-PO cells with 20 nM Aplidin for 1 h and tested the medium of the cells by means of an ELISA assay used to measure VEGF-A levels of secretion at 0, 6 and 24 h after drug-washout. As depicted in Figure 9

Aplidin was able to substantially abolish VEGF secretion in the medium at 6 and 24 h after drug-washout.

### Cytotoxicity and apoptosis induced by Aplidin on primary ALL cells

To assess the cytotoxic effect of Aplidin on fresh leukaemia cells obtained directly from patients affected by BCP-ALL, a stroma-supported immunocytometric assay was used. Previous studies have shown that, under these culture conditions, phenotypic and karyotype features of leukaemia cells are maintained even after several months of culture.

Table 1 shows the clinical features of the ALL patients at the time when the cells were derived. In nine out of 14 cases (nos. 1–9) the leukaemia cells were collected at the time of diagnosis, while in the other five cases (nos. 10–14), at the time of relapse. Among the 14 samples of ALL studied, the number of cells recovered after 7 days of stromal-supported culture ranged from 38 to 210% (median 83%) of those originally seeded.

**Figure 10:**
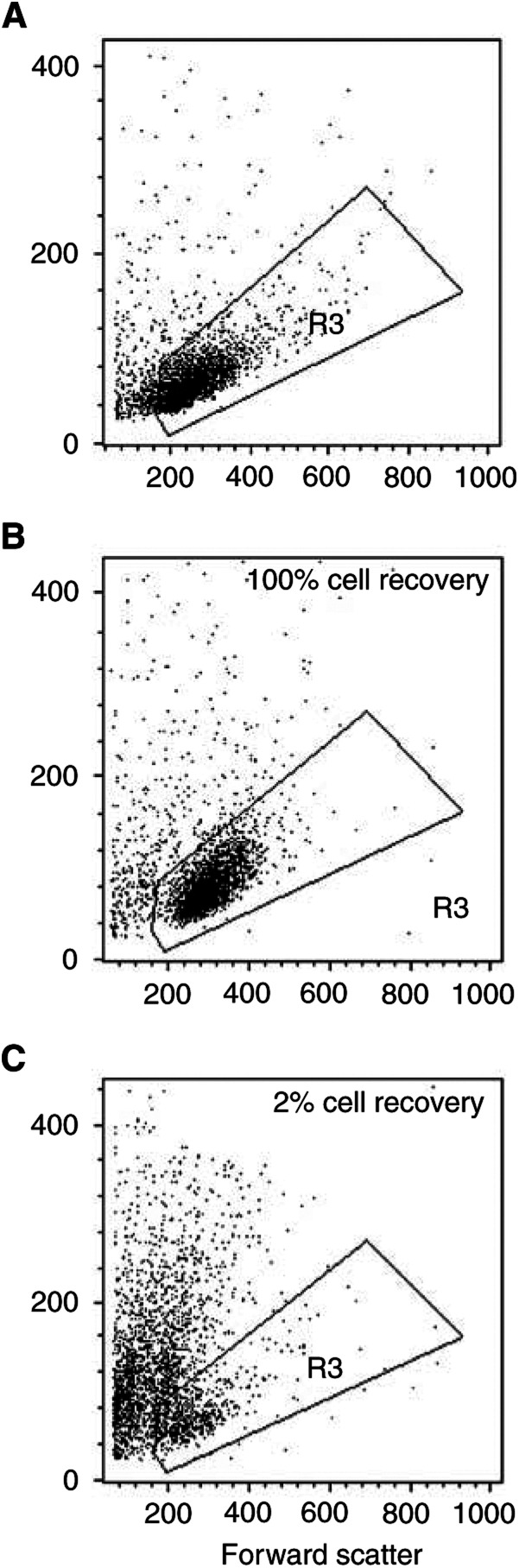
Example of light-scatter dot-plot analysis of blast cells growing on stroma feeder layer evaluated by flow cytometry. Forward and side scatter analysis were evaluated at the beginning of culture (**A**), after 7 days of culture (**B**, control cells) and after 7 days with 5 nM Aplidin (**C**, treated cells).

It is known that changes in forward/side light-scatter parameters, consisting in a reduction in forward scatter, indicating a reduction in cell size, and an increase in side scatter, indicating an increase in cell granularity, are frequently associated with apoptosis. As detected on leukaemic cell lines Aplidin induced changes in the light-scattering properties by using flow cytometry forward/side light-scatter analysis on ALL cells too, suggesting an induction of apoptosis (Figure 10

shows a representative case).

As shown in Table 2

**Table 2:** Stroma-supported immunocytometric assay % cell death after 7 days Aplidin exposure

	**Aplidin (nM)**			
**Patient no**	**0.005 nM**	**0.05 nM**	**0.05 nM**	**5 nM**
1	NT	12	22	96
2	NT	79	79	100
3	14	20	40	99
4	16	36	60	100
5	12	19	46	100
6	<1	<1	9	96
7	<1	<1	<1	74
8	<1	<1	<1	97
9	<1	<1	<1	90
10^a^	31	3	35	82
11^a^	<1	1	49	98
12^a^	10	9	99	88
13^a^	47	33	55	97
14^a^	<1	ND	50	97

a =relapse. ND=not determined.

, the cytotoxic effect of Aplidin on BCP-ALL cells was dose dependent. Aplidin at the concentration of 5 nM was strongly cytotoxic in all cases. The percentage of cell death ranged from 74 to 100% (median 97%). At the concentration of 0.5 nM Aplidin cell killing effect was not detectable in three cases (nos. 7, 8 and 9), while in the remaining 11 cases it ranged from nine to 99% (median 49%). Aplidin at 0.05 nM induced cell death in seven out of 13 cases (nos. 1–5 12 and 13) with a range from nine to 79% (median 20%). At 0.005 nM substantial cytotoxicity (47% compared to control cells) was observed only in one case (no. 13). Interestingly, the same level of cell kill (median 97%) was obtained with 5 nM Aplidin in cells taken either at the time of diagnosis (nos. 1–9) or at relapse (nos. 10–14).

The cytotoxicity of Aplidin extended to sample nos. 7 and 9, each of which carries adverse genetic features such as t(9;22) and t(4;11), respectively.

To investigate whether Aplidin cytotoxicity represents a direct effect on leukaemic cells or an indirect effect mediated by damage of the stroma layers, we incubated the stroma layers for 7 days with different Aplidin concentrations ranging from 0.005 to 5 nM. Then the cells were washed and seeded with leukaemic lymphoblasts in drug-free medium for 7 days. We found that the morphology and cell confluence were not affected by Aplidin treatment. In four out of five cases the percentage cell recovery obtained after 5 nM Aplidin exposure was between 78 and 92%. Only in one case did the ability of stromal cells to support leukaemia cell survival after Aplidin treatment not occur (data not shown).

## DISCUSSION

This study shows that Aplidin is a potent antileukaemic agent against human lymphoblastic leukaemia cell lines, as well as fresh leukaemia cells derived directly from patients with childhood BCP-ALL. We found that on Philadelphia chromosome-positive TOM-1 and ALL/MIK with t(9;22), on ALL-PO with t(4;11) and on Reh t(12;21) cell lines, Aplidin induced a strong growth inhibition effect at nanomolar concentrations, the IC_50_ ranging from 5 to 20 nM.

The present study also shows that Aplidin is a strong inducer of apoptosis, a finding in keeping with previous data obtained on another human leukaemia Molt-4 cell line (Erba *et al*, 2002; Broggini *et al*, 2003). In all the cell lines investigated, the Aplidin-induced cell death was clearly related to the induction of apoptosis, even if in Reh cells the apoptosis was found only when the cells were exposed to high concentrations of Aplidin. ALL/MIK and TOM-1 cells express wt p53, whereas the other a mutated p53 (M Broggini, personal communication), thus indicating that Aplidin can induce apoptosis in a p53-independent manner.

We have recently reported that in Molt-4 cells Aplidin causes an inhibition of VEGF secretion and a downregulation of flt-1 (Broggini *et al*, 2003). The apoptosis induced by Aplidin in Molt-4 cell could be observed also by exposing the leukaemic cells to anti-VEGF antisense oligonucleotide (Gerber *et al*, 1998; Nor *et al*, 1999; Broggini *et al*, 2001, 2003). In these cells, the addition of VEGF could antagonise the apoptotic process induced by Aplidin. These data suggested that a potential mechanism of cytotoxicity of Aplidin was related to the blocking of an autocrine loop relevant for cell growth and survival. In the present study, however, we could not confirm this finding. In only one cell line, that is ALL-PO cells, we found evidence of the same phenomenon previously seen in Molt-4 cells. Since the sensitivity of ALL-PO cells to Aplidin is comparable to that observed for other ALL cell lines such as Reh, ALL/MIK and TOM-1, in which Aplidin was not inducing any effect on VEGF secretion, it should be concluded that Aplidin cytotoxicity against ALL cells is not related to VEGF inhibition. It may be speculated, however, that in the cell lines in which no block of VEGF loop was observed, Aplidin cytotoxicity is mediated by the inhibition of other growth factors. The evidence that Aplidin induces changes in the expression of genes involved in different cellular pathways (Marchini *et al*, 2002) is in line with this hypothesis. However, these aspects require to be further investigated.

The available data on the effects of didemnins on the cell cycle consistently indicate that these compounds cause a block of the cells in G_1_ (Crampton *et al*, 1984; Erba *et al*, 2002). It has been recently reported that in Molt-4 Aplidin mainly induces a G_1_ block, but a more sophisticated analysis revealed that the drug induced a G_2_ block too (Erba *et al*, 2002). In particular, by using a simulation program (Montalenti *et al*, 1998) suitable to describe drug-induced cell cycle block, delay, repair and death effects, it became apparent that a G_2_ blockade also occurs in Molt-4 cells exposed to Aplidin. In the present study we can confirm that in addition to a G_1_ arrest a G_2_ blockade was induced by Aplidin in ALL cell lines.

Aplidin was found to induce a strong cytotoxicity in patient-derived leukaemia cells evidenced in a stroma-supported immunocytometric assay. This system has been successfully used to investigate the antileukaemic activity of different compounds, such as 2-chloro-2-deoxyadenosine (Kumagai *et al*, 1994), Interferon-*α* (Manabe *et al*, 1993), Cyclosporin A (Ito *et al*, 1998), Taxol and Taxotere (Consolini *et al*, 1998), Vincristine, Teniposide and Ara C (Campana *et al*, 1993, 1999). Aplidin at 5 nM caused a dramatic cell death (median 97%) in all the 14 cases studied. At 0.5 nM cell death was still present in 11 out of 14 cases (median 49%). Although in some cases there might be discrepancies between the *in vitro* cytotoxic concentration and the active anticancer drug plasma levels, note that the Aplidin concentrations used are pharmacologically reasonable as Aplidin concentrations above 20 nM are achievable for several hours in the plasma of patients receiving the drug given as 24 h in a range doses much lower than the maximum tolerated dose of 6000 *μ*g m^−2^ (Zucchetti, personal communication).

Cells from two children with genetic abnormalities such as t(9;22) and t(4;11) translocation, which are associated with an inferior treatment outcome, were sensitive to Aplidin to the same extent as that observed in other BCP-ALL cases. Likewise, the cell lines with t(9;22) (ALL/MIK and TOM-1) or t(4;11) (ALL-PO) were strongly sensitive to Aplidin at similar concentrations.

In relapsed ALL cases, Aplidin exerted a strong cell killing effect (97%) in all five primary cells indicating that Aplidin is a candidate antileukaemic agent in patients with ALL that are nonresponsive to standard chemotherapeutic agents.

The data obtained with ALL cell lines and on Molt-4 cells (Erba *et al*, 2002) clearly indicate a direct antileukaemic activity of Aplidin. However, in the stroma-supported cultures of BCP-ALL cells derived from patients, the Aplidin-induced apoptosis could be due to a toxic effect to stroma cells (Campana *et al*, 1993; Consolini *et al*, 1998; Ito *et al*, 1998). We did not find a decrease in the capacity of stroma pretreated with Aplidin, to support ALL cell viability. Recently (Albella *et al*, 2002), similar data have been reported on human bone haematopoietic progenitors treated by Aplidin. At concentrations similar to those used in this study Aplidin did not induce growth inhibition in the tested haematopoietic progenitors by using clonogenic assay. It must be taken into account that stroma is characterised by the presence of different cell types including endothelial, reticulo cells, macrophages, fibroblast and adipocytes. As the stroma layers used in this study were derived from different patients, the reduced survival of ALL cells found in one case after exposure to 5 nM Aplidin, could be related to biological variability in the susceptibility of the different cell types present in the stroma layer.

Although the treatment outcome of children affected by ALL showed marked improvements in the last decade, in one-third of the children, ALL is fatal. Identification of new antileukaemia agents is essential for improving the survival of patients with high-risk or refractory leukaemia. Clinical Phase I and II studies of Aplidin have shown antitumour activity in patients with neuroendocrine tumours and medullary thyroid carcinomas (Raymond *et al*, 2000; Armand *et al*, 2001; Ciruelos Gil *et al*, 2002). Since at the recommended doses for phase II studies Aplidin plasma levels are maintained for many hours in the range of 10–100 nM (Zucchetti, personal communication; Maroun *et al*, 2001, according to the results presented in this study it seems realistic to assume the drug has a potential for therapy of ALL patients resistant to or relapsing from the available chemotherapies.
